# Serologic Evidence of Frequent Human Infection with WU and KI Polyomaviruses

**DOI:** 10.3201/eid1508.090270

**Published:** 2009-08

**Authors:** Nang L. Nguyen, Binh-Minh Le, David Wang

**Affiliations:** Washington University in St. Louis, St. Louis, Missouri, USA

**Keywords:** WU polyomavirus, KI polyomavirus, seroepidemiology, viruses, research

## Abstract

WU polyomavirus (WUPyV) and KI polyomavirus (KIPyV) are novel human polyomaviruses. They were originally identified in human respiratory secretions, but the extent of human infection caused by these viruses has not been described to date. To determine the seroepidemiology of WUPyV and KIpyIV, we used an ELISA to screen serum samples from 419 patients at the St. Louis Children’s Hospital and Barnes-Jewish Hospital during 2007–2008. The age-stratified deidentified samples were examined for antibodies to the major capsid proteins of WUPyV and KIPyV. Seropositivity for each virus was similar; antibody levels were high in the youngest age group (<6 months), decreased to a nadir in the next age group (6 to <12 months), and then steadily increased with subsequent age groups, eventually reaching a plateau of ≈80% for WUPyV and ≈70% for KIPyV. These results demonstrate that both KIPyV and WUPyV cause widespread infection in the human population.

## Abstract 2

WU and KI polyomavirus infections are widespread.

WU polyomavirus (WUPyV) (*1*) and KI polyomavirus (KIPyV) (*2*) are newly described human polyomaviruses most closely related to JC virus (JCV) and BK virus (BKV). JCV and BKV are human pathogens that commonly infect the population. In the United States, seropositivity rates of 44%–75% for JCV and 63%–80% for BKV have been reported (*3*,*4*). Current models suggest that initial infection by BKV and JCV occurs asymptomatically during childhood; latency may establish in the kidneys and may reactivate during immune suppression. JCV causes a fatal demyelinating disease of progressive multifocal leukoencephalopathy in immunocompromised persons (*5*). BKV is associated with a number of renal and urinary tract infections including tubular nephritis, which can lead to allograft failure in renal transplant recipients (*6*), and hemorrhagic cystitis in hematopoietic stem cell transplant recipients (*7*). Another human polyomavirus, Merkel cell polyomavirus, was recently discovered and has tentatively been linked to Merkel cell carcinoma (*8*).

For KIPyV and WUPyV, neither disease association nor extent of infection in the human population has been established. Both viruses were originally identified in specimens from patients with respiratory illnesses of unknown etiology. Subsequent studies found WUPyV and KIPyV in the respiratory tract of patients with and without respiratory signs and symptoms (*9*–*11*), in fecal samples (*12*,*13*), and in lymphoid tissue from immunocompromised persons (*14*). Reported prevalence rates are 1%–9% for WUPyV and 0.5%–3% for KIPyV (*1*,*2*,*12*,*13*). The severity of diseases caused by BKV and JCV (*5*,*6*,*15*) raises the question of whether WUPyV and KIPyV can cause human disease. As a step toward determining the potential pathogenicity of these viruses, we developed serologic assays to assess the extent of infection by WUPyV and KIPyV in humans.

## Materials and Methods

### Plasmid Constructs, Protein Expression, and Purification

Genes encoding the major capsid proteins, KIPyV viral protein 1 (VP1) and WUPyV VP1, were cloned into the Gateway vector pENTR/SD/D-TOPO (Invitrogen, Carlsbad, CA, USA) by PCR from clinical samples. The primers were as follows: 5′-CACCATGAGCTGCACCCCGT-3′ (forward) and 5′-ATACATTCACTTTGAATTTTGTTGAG-3′ (reverse) for the KIPyV VP1 PCR and 5′-CACCATGGCCTGCACAGCAAAGCCAGCC-3′ (forward) and 5′-TTATCCTTGTGTGTTTAGTATTGG-3′ (reverse) for the WUPyV VP1 PCR. Sequencing analysis showed that the KIPyV VP1 gene cloned was identical to that of the Brisbane 002 strain (GenBank accession no. ABR68682), except for 2 silent nucleotide mutations at positions 537 and 1005. For WUPyV, the gene encoding VP1 was identical to that of the B0 strain (GenBank accession no. ABQ09289). Positive clones containing the inserts were then transferred into the p-DEST15 plasmid (Invitrogen) by LR-homologous recombination to generate N-terminal–tagged glutathione S-transferase (GST)–WUPyV VP1 (plasmid NN003) and GST-KIPyV VP1 (NN006) constructs. N-terminal–tagged GST-VP1s from BKV, JCV, and simian virus 40 (SV40) were generously provided by Michael Pawlita (*16*), and GST-tagged microneme (Mic) protein encoded by *Toxoplasma gondii* was provided by David Sibley. VP1 was expressed in BL21(DE3)pLysS bacterial cells and affinity purified under native conditions by using the BugBuster GST-Bind Purification Kit (Novagen, Darmstadt, Germany) according to the manufacturer’s suggested protocol.

### Polyacrylamide Gel Electrophoresis and Western Blot Analysis

Proteins were separated by electrophoresis in 4%–15% polyacrylamide gradient gels (no. 161-1122; BioRad, Hercules, CA, USA) by using Tris/glycine/sodium dodecyl sulfate (SDS) buffer (no. 161–0732; BioRad). The proteins were then either stained with Coomassie brilliant blue or transferred to a polyvinylidene difluoride membrane (no. LC2002; Invitrogen) for Western blot immunoassay. Membranes were blocked with 5% nonfat milk in phosphate-buffered saline with Tween 20 (PBS-T) for 1 h, then incubated with the primary antibody followed by peroxidase-conjugated Protein A/G (no. 32490; Pierce Biotechnology, Rockford, IL, USA). The proteins were visualized by using a SuperSignal West Pico kit (no. 34077; Thermo Scientific, Rockford, IL, USA). Membranes that were probed >1× were stripped with Restore Western Blot Stripping Buffer (no. 21059; Thermo Scientific) and reblocked with 5% nonfat milk in PBS-T between immunoassays.

### Antibody Production

WUPyV VP1 peptide sequence (TAKPGRSPRSQPTRC) and KIPyV VP1 peptide sequence (CRPQKRLTRPRSQV) were each synthesized and injected into rabbits to produce polyclonal antibodies against WUPyV VP1 and KIPyV VP1 (service provided by GenScript, Piscataway, NJ, USA). Rabbit hyperimmune antiserum against the virus-like particles of BKV (BKVLP), JCV (JCVLP), or SV40 were kindly provided by Joakim Dillner (*17*). Peroxidase-conjugated goat anti-human immunoglobulin (Ig) G, (no. 31413) peroxidase-conjugated goat anti-rabbit IgG (no. 31463), and mouse anti-GST (no. 30001) antibodies were obtained from Thermo Scientific.

### Serum Sample Analyses

We analyzed 419 deidentified serum samples from patients 1 day to 79 years of age at St. Louis Children’s Hospital or Barnes-Jewish Hospital in St. Louis, Missouri, USA, from November 2007 through October 2008 for antibodies against VP1 of WU and KI polyomaviruses. Serum samples were kindly provided by Greg Storch (St. Louis Children’s Hospital) and Mitchell Scott (Barnes-Jewish Hospital). The patients were age stratified (Table), and 30 samples were used for each age group, except for the group 6 to <12 months, for which 29 samples were available. Collection of samples and clinical data were approved by the Human Research Protection Office of Washington University in St. Louis, School of Medicine.

**Table:** Age distribution of patients (419 samples) who were seropositive for WUPyV, KIPyV, or both, St. Louis, Missouri, USA, November 2007–October 2008*

Age group	No. (%) seropositive		
	KIPyV VP1	WUPyV VP1	Both
<6 mo	13 (43.3)	25 (83.3)	13 (43.3)
6 mo–<1 y	7 (24.1)	13 (44.8)	7 (24.1)
1 y	12 (40)	18 (60)	10 (33.3)
2 y	13 (43.3)	18 (60)	10 (33.3)
3 y	15 (50)	17 (56.7)	10 (33.3)
4 y	22 (73.3)	20 (66.7)	16 (53.3)
5 y	28 (93.3)	26 (86.7)	24 (80)
6–8 y	26 (86.7)	27 (90)	24 (80)
9–12 y	30 (100)	27(90)	27 (90)
13–19 y	28 (93.3)	28 (93.3)	27 (90)
20–34 y	21 (70)	30 (100)	21(70)
35–49 y	22 (73.3)	29 (96.7)	22 (73.3)
50–64 y	19 (63.3)	24 (80)	19 (63.3)
65–79 y	22 (73.3)	28 (93.3)	22 (73.3)
Total	278 (66.3)	330 (78.7)	252 (60.1)

*WUPyV, WU polyomavirus; KIPyV, KI polyomavirus; WUPyV VP1, WUPyV viral protein 1; KIPyV VP1, KIPyV viral protein 1.

### ELISA

To develop the WU ELISA and the KI ELISA, we used as positive controls 2 convalescent serum samples from a child known to be infected with WUPyV and rabbit hyperimmune serum for WUPyV VP1 or KIPyV VP1, and we used as negative controls rabbit preimmune serum and serum derived from pediatric patients (<3 years of age). The optimal coating concentration of VP1, serum dilution, secondary conjugate dilution, and blocking reagent were determined by checkerboard titration experiments. Briefly, purified GST-VP1 (0.12 μg/well) was coated overnight at 4°C in PBS, pH 7.2, by using Maxisorp 96-well microtiter plates (Nunc, Naperville, IL, USA). Wells were washed 3× with PBS containing 0.05% PBS-T and blocked with 2% nonfat milk in PBS-T (PBS-TM) for 2 h at room temperature. Human serum samples (60 μL), diluted 1:100 in PBS-TM, were added to each well in triplicate and incubated for 4 h (or overnight) at 4°C. The plates were washed 6× with PBS-T, and a peroxidase-conjugated, secondary anti-IgG antibody, diluted 1:40,000 in PBS-TM, was added and incubated at 37°C for 2 h. After another washing step, plates were developed by adding peroxidase substrate tetramethyl benzidine (no. 34028; Thermo Scientific) for 15 min at room temperature. The reactions were stopped with H_2_SO_4_, and the absorbance was measured at 450 nm. For the blocking assays, serum samples were preincubated overnight at 4°C with, either singly or together in different combinations as indicated, recombinant GST-BKVP1, GST-JCVP1, GST-KIPyV VP1, GST-SV40VP1, GST-WUPyV VP1, or an unrelated GST-Mic encoded by *Toxoplasma gondii* (at 0.6 μg each, in solution), or in the blocking buffer alone. The ELISA was then used as described above.

### Cutoff Value and Statistical Analysis

To calculate a cutoff value for the WU ELISA, we used 31 pediatric serum samples that gave signals below that of rabbit preimmune serum. Samples with absorbance intensity >3 SDs above the mean of these 31 samples (0.404 ± 0.103 SD) were considered positive. A parallel set of 31 negative samples (mean 0.286 ± 0.095 SD) were used to calculate a cutoff value for the KI ELISA. For each WU ELISA 96-well plate, the same negative control sample (serum from a 3-month-old child previously considered negative by initial ELISA experiments) and the same positive control sample (convalescent-phase serum from a patient previously found to be WU positive) were used to control for interplate variations. The cutoff value for percentage coefficient of variation of these 2 control samples was set <30%, as described by Jacobson (*18*). All blank wells had absorbance values <0.1.

## Results

### WUPyV VP1 and KIPyV VP1 Proteins as Target Antigens in ELISAs

**Figure 1:**
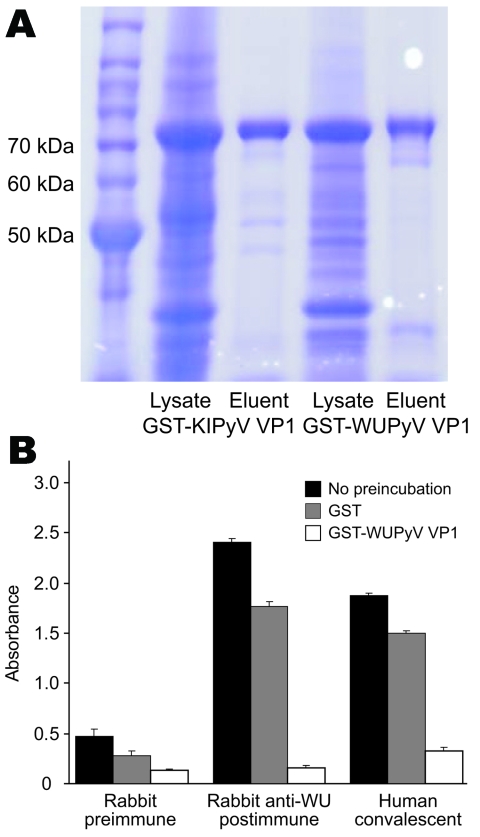
ELISA using WU polyomavirus (WUPyV) viral protein 1 (VP1) or KI polyomavirus (KIPyV) VP1 as the target antigen. A) Coomassie blue staining of a sodium dodecyl sulfate–polyacrylamide gel that contains bacterially expressed glutathione S-transferase (GST)–KIPyV VP1 and GST–WUPyV VP1 before and after glutathione-affinity purification. B) ELISA using rabbit hyperimmune serum and human WU polyomavirus convalescent-phase serum preincubated with buffer alone, GST protein, or GST–WUPyV VP1. Error bars indicate mean and SD.

WUPyV VP1 and KIPyV VP1 were expressed in bacteria as N-terminal, GST-tagged fusion proteins and subsequently purified by using glutathione-affinity chromatography. We used SDS polyacrylamide gel electrophoresis coupled with Coomassie blue staining to analyze the production and purification of the recombinant proteins (Figure 1, panel A). The purified GST-WUPyV VP1 or GST-KIPyV VP1 was then used as the capture antigen in ELISA to detect antibodies against WUPyV VP1 or KIPyV VP1, respectively.

The results of a WU ELISA using WU-hyperimmune rabbit serum and WU-positive human convalescent-phase serum are shown in Figure 1, panel B. Both the rabbit and human serum samples gave strong signals, which were effectively inhibited by preincubation with soluble GST-WUPyV VP1. By contrast, preincubation with GST alone had only marginal effects on the ELISA signal intensity. An ELISA performed on a GST-KIPyV VP1 coated plate using KI-hyperimmune rabbit serum also showed similar KI-specific binding activity (data not shown).

### Detection of Antibodies against WUPyV VP1 and KIPyV VP1 in Human Serum Samples

**Figure 2:**
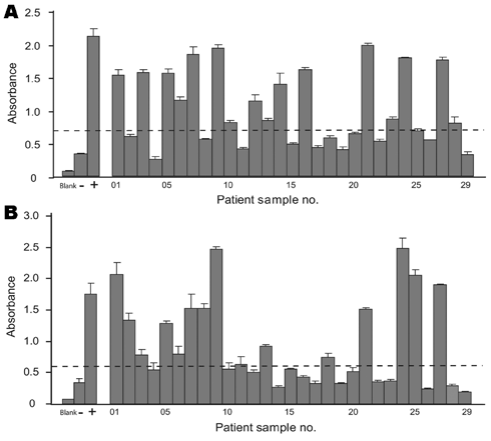
ELISA results from 3-year age group. A) 29 serum samples in the 3-year age group were assayed for antibodies against WU polyomavirus viral protein 1. B) KI polyomavirus ELISA results from the same 29 serum samples. The cutoff values for WU ELISA (0.713) and KI ELISA (0.572) are represented by dashed lines; –, negative control serum; +, positive control serum. Error bars indicate mean and SD.

Of the 419 serum samples analyzed, a representative WU ELISA result of 29 serum samples in the 3-year age group is shown in Figure 2, panel A. A range of absorbance intensities were observed; 17 samples were above the cutoff value. In a parallel KI ELISA conducted on this same set of serum samples, using rabbit serum immunized with a synthetic KIPyV VP1 peptide as positive control, 15 samples were considered ELISA positive (Figure 2, panel B).

### Specificity of VP1-based WU ELISAs and KI ELISAs

**Figure 3:**
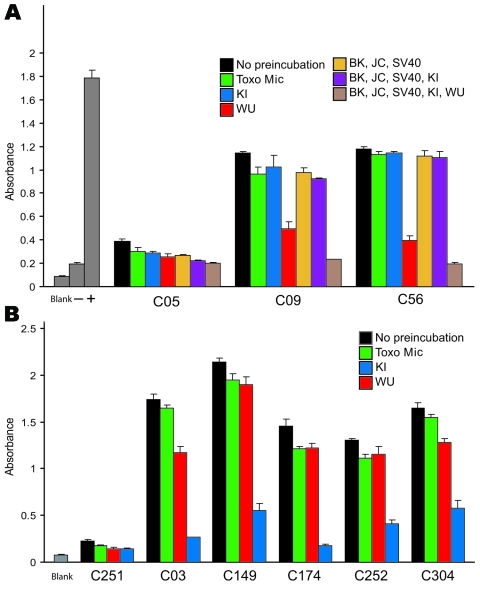
Effects of preincubation of polyomavirus viral protein 1 on WU ELISA and KI ELISA. Samples were preincubated with single or multiple proteins in different combinations or with the blocking buffer alone. Shown are results of blocking WU ELISA (A) and blocking KI ELISA (B) of representative serum samples. Proteins used in preincubation experiments are indicated by matching color scheme. Blocking data for WU ELISA–negative serum (C05) and KI ELISA–negative serum (C251) are shown for comparison. Toxo Mic, microneme protein from *Toxoplasma gondii*; SV40, simian virus 40. Error bars indicate mean and SD.

WUPyV VP1 shares 65% amino acid identity with KIPyV VP1 and more limited similarity with JCVP1 (27%), BKVP1 (28%), and SV40VP1 (28%) (*1*). Previous serologic studies demonstrated that antibodies against VP1 from BKV, JCV, and SV40 polyomaviruses were cross-reactive with antigens from all 3 of these viruses (*4*,*19*). To assess the specificity of the WU ELISA and KI ELISA, we expressed and purified GST-tagged VP1 from BKV, JCV, and SV40 and performed blocking ELISAs as described above. Figure 3, panel A, shows a blocking WU ELISA of 3 serum samples that had been preincubated with, either singly or together in different combinations, GST-BKVP1, GST-JCVP1, GST-KIPyV VP1, GST-SV40VP1, and GST-WUPyV VP1; with an unrelated GST-Mic protein; or with the blocking buffer alone. Two WU-positive samples, C09 and C56, had absorbance values reduced to some extent after preincubation with different recombinant proteins. However, only when preincubated with the GST-WUPyV VP1 itself was the ELISA signal intensity strongly reduced; C56 was reduced by 83.5% and C09 by 79.6% compared with the buffer-alone samples. Of 34 randomly selected WU ELISA–positive samples tested, 32 showed inhibition levels >50% in the presence of soluble GST-WUPyV VP1. The 2 exceptions, A04 and A43, had limited inhibition of 42.5% and 36%, respectively. For these 2 samples, further dilution of the human serum enabled soluble GST-WUPyV VP1 to reduce the ELISA absorbance intensity by >50%, suggesting perhaps that high titers of antibodies against WUPyV VP1 were present in these 2 samples (data not shown). To define the specificity of the KI ELISA, we preincubated ELISA-positive samples with soluble GST-Mic, GST-WUPyV VP1, GST-KIPyV VP1, or the buffer alone. Figure 3, panel B, shows blocking results of 5 representative KI-positive samples. In each example, only preincubation with soluble GST-KIPyV VP1 substantially reduced the absorbance value, indicating the presence of specific antibodies against KIPyV VP1 in these samples.

### Detection of Antibodies against WUPyV VP1 and KIPyV VP1 by Western Blot

**Figure 4:**
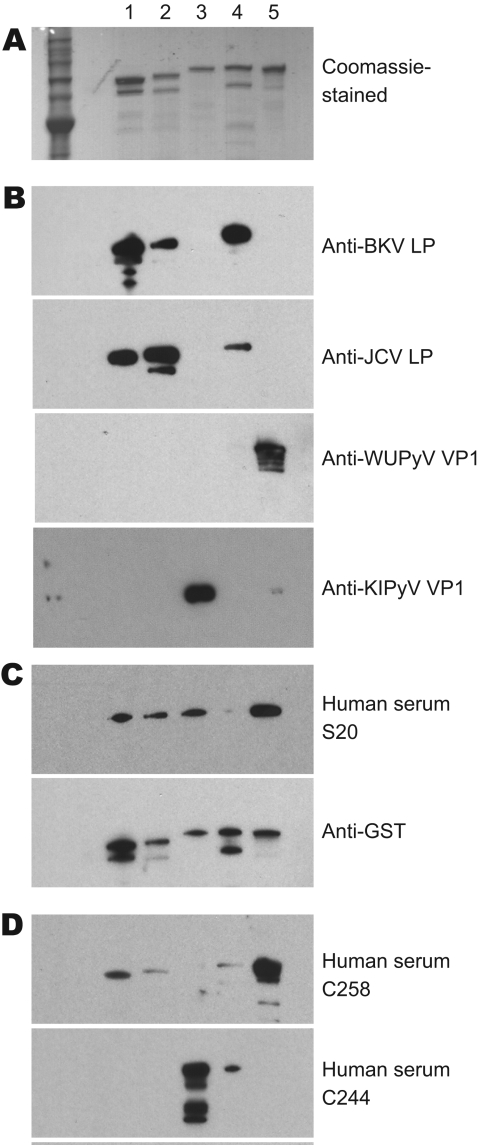
Results of patient serum sample Western blotting for polyomaviruses. A) Coomassie blue–stained image showing 5 types of purified glutathione S-transferase (GST)–tagged viral protein 1 (VP1) in a sodium dodecyl sulfate–polyacrylamide gel. Lane 1, GST-BKV VP1; lane 2, GST-JCV VP1; lane 3, GST–KI polyomavirus (KIPyV) VP1; lane 4, GST-SV40 VP1; lane 5, GST–WU polyomavirus (WUPyV) VP1. B) Western blot results using control rabbit antiserum against BK virus-like particles (BKVLP), JC virus-like particles (JCVLP), WUPyV VP1, or KIPyV VP1 as primary antibody. C) Western blot results for serum that was positive (S20) for WU polyomavirus and KI polyomavirus by ELISA. Antibody against GST was used as a loading control. D) Western blot result for serum that was ELISA positive for WU (C258) and KI (C244).

To confirm the results of our WU ELISAs and KI ELISAs and further assess the potential serum cross-reactivity with VP1 proteins of related polyomaviruses, we performed Western blot assays. The affinity purified GST-BKVP1 (69.3 kDa), GST-JCVP1 (68.8 kDa), GST-KIPyV VP1 (70.7 kDa), GST-SV40VP1 (69.3 kDa), and GST-WUPyV VP1 (69.0 kDa), which all migrated to their relatively expected position in an SDS-polyacrylamide gel (Figure 4, panel A), were used as target antigens.

Hyperimmune rabbit serum against BKVLP reacted strongly with BKVP1 and SV40VP1 and, to a lesser extent, with JCVP1. Anti-JCVLP rabbit serum reacted strongly to both JCVP1 and BKVP1 but rather weakly to SV40VP1. These results were consistent with those of previous studies describing cross-reactivity among the VP1 of BKV, JCV, and SV40 (*19*). Neither rabbit serum control, however, showed any cross-reactivity to WUPyV VP1 or to KIPyV VP1 (Figure 4, panel B).

A set of human serum samples was randomly selected for confirmatory Western blotting. Figure 4, panels C and D, shows the results of 3 representative samples. They included a sample that was positive by ELISA for antibodies against WUPyV VP1 and KIPyV VP1 (S20), a sample that was ELISA positive for WUPyV but negative for KIPyV (C258), and a sample that was positive by ELISA for KIPyV and negative for WUPyV (C244). In each case, Western blot analysis corroborated the ELISA results.

### Seroprevalence Rates

**Figure 5:**
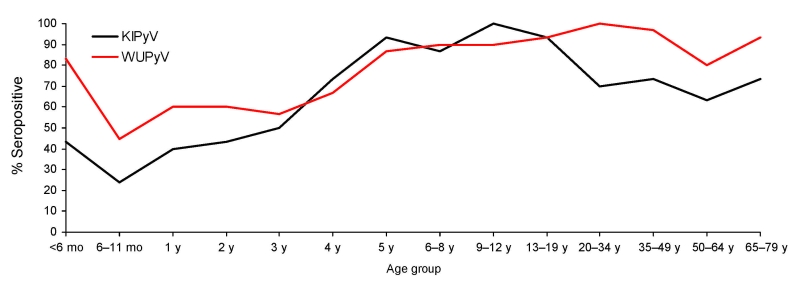
Percentage of serum samples positive for antibodies against WU polyomavirus (WUPyV) and KI polyomavirus (KIPyV), by patient age group.

Overall WUPyV seropositivity in this cohort was 78.7% (330/419), KIPyV seropositivity was 66.3% (278/419), and seropositivity for both viruses was 60.1% (252/419) (Table). In general, the rate of WUPyV seropositivity was slightly higher than KIPyV seropositivity throughout. Antibodies against WUPyV and KIPyV appeared to be maternally transmittable, on the basis of the high degree of seropositivity detected in the <6-month age group. The lowest seropositivity rates were observed for the age group 6 to <12 months (44.8% for WUPyV and 24.1% for KIPyV), consistent with the waning of maternal antibody levels. Infection rates for both viruses then increased rapidly, reaching 86.7% for WUPyV and 93.3% for KIPyV in the 5-year age group. For patients >6 years of age, average WUPyV seropositivity was 80% (193/240). In this dataset, KIPyV infection appeared to peak in the age group 5–19 years (average seropositivity rate 93%), but in patients >20 years of age, the average was 70% (Figure 5).

## Discussion

We developed WUPyV-specific and KIPyV-specific ELISAs to assess the extent of human infection by WUPyV and KIPyV. Previous studies on BKV, JCV, and SV40 polyomaviruses indicated that the viral major capsid protein is immunodominant and that human serum can recognize the recombinant VP1 expressed in bacteria (*16*). We demonstrate that the full-length recombinant WUPyV VP1 and KIPyV VP1 expressed in bacteria are capable of detecting antibodies against WUPyV and KIPyV VP1, respectively, using either the ELISA or the Western blot assay format.

One issue that has confounded serologic analysis of human polyomaviruses in the past is antigenic cross-reactivity (*4*,*19*). We performed a number of experiments to define whether cross-reactivity exists between antibodies against WUPyV VP1 in human serum and the VP1 antigens of KIPyV, SV40, BKV, and JCV. Preincubation experiments demonstrated that the GST-VP1 from BKV, JCV, and SV40 reduced the absorbance signals only minimally, equivalent to the reduction observed when an irrelevant GST-fusion protein, GST-Mic, was used (Figure 3, panel A). These data indicate that BKV, JCV, and SV40 VP1s are not cross-reactive with antibodies against WUPyV VP1. This observation was further substantiated by Western blotting using rabbit serum against virus-like particles of BKV and JCV, which yielded no detectable cross-reaction with WUPyV VP1 or KIPyV VP1. Because WUPyV VP1 and KIPyV VP1 share 65% sequence identity, potential cross-reactivity between them was a concern. However, preincubation with GST-KIPyV VP1 reduced the WU ELISA absorbance signal by only 13.5% on average, compared with 10.4% on average when an irrelevant GST protein or VP1 of BKV, JCV, and SV40 were used. Similarly, KI ELISA showed an average of 16.9% and 9.4% signal reduction when samples were preincubated with GST-WUPyV VP1 or GST-Mic protein, respectively.

As a separate line of evidence, Western blotting with serum that was positive for antibodies against WUPyV VP1 and negative for antibodies against KIPyV VP1 (C258) and serum that was negative for WUPyV VP1 and positive for KIPyV VP1 (C244) yielded no evidence of cross-reactivity (Figure 4, panel D). Collectively, the ELISA preincubation experiments and the Western blotting demonstrated minimal, if any, detectable cross-reactivity between KIPyV and WUPyV within the sensitivity limits of these assays.

Our findings demonstrate high sustained rates of infection by WUPyV and KIPyV in this cohort. Although the serum samples were selected by using broad inclusion criteria (specimen volume >0.5 mL) from patients visiting St. Louis Children’s Hospital and Barnes-Jewish Hospital, both tertiary referral hospitals, these results may be somewhat biased toward patients with some form of disease. Additional experiments with patient cohorts from different regions or with different inclusion criteria are necessary to generalize these results to the general population. The steep increase in seropositivity for WUPyV and KIPyV in patients 4–6 years of age raises the possibility that school attendance plays a role in facilitating transmission of these viruses. Serologic studies of other viruses such as bocavirus and BKV also showed elevated rates of infection in children of similar age (*20*–*22*). For KIPyV, the observed decrease in seropositivity rate for patients >19 years of age raises the possibility that antibodies against KIPyV wane over time. However, direct experimentation using longitudinally collected serum would be necessary to address this possibility.

In conclusion, we have established WUPyV-specific and KIPyV-specific ELISAs using VP1 as the capture antigen. We have demonstrated that cross-reactivity between antibodies against WUPyV VP1 (or KIPyV VP1) and VP1 from KIPyV (or WUPyV), BKV, JCV, or SV40 viruses is minimal. We also demonstrated that WUPyV and KIPyV cause widespread infection in humans. These observations roughly parallel the extent of human exposure to BKV and JCV. Given that severe illnesses have been associated with infections by BKV and JCV, further studies are needed to determine whether WUPyV and KIPyV are similarly pathogenic.
